# Krill's Disease: A Newer Management Option

**DOI:** 10.18502/jovr.v18i3.13782

**Published:** 2023-07-28

**Authors:** Sarang P Lambat, Vinay B Nangia, Prabhat V Nangia, Neha H Shah, Swati D Mishra

**Affiliations:** ^1^Vitreoretinal Services, Suraj Eye Institute, Nagpur, India; ^2^Cornea and Refractive Services, Suraj Eye Institute, Nagpur, India

**Keywords:** Acute Retinal Pigment Epithelitis, Intravenous Methylprednisolone, Krill's Disease

## Abstract

**Purpose:**

To report a case of a young female who presented with scotoma in the right eye for few days.

**Case Report:**

Krill's disease or acute retinal pigment epithelitis (ARPE) is a self-limiting retinal disease with no specific treatment. Typical clinical and imaging features helped us to diagnose her with ARPE. Intravenous methylprednisolone (IVMP), which gives a rapid anti-inflammatory response, was advised. An SD-OCT scan post-injection showed a reduction in hyperreflectivity and height of lesion at day 3 and near total resolution by day 5.

**Conclusion:**

This case suggests rapid resolution of ARPE with the use of IVMP.

##  INTRODUCTION

Acute retinal pigment epithelitis (ARPE) is a rare, idiopathic, self-limiting inflammatory disease of the retina that commonly affects young adults.^[1,2]^ It was first described in 1972 by Alex E. Krill and August F. Deutman. They described these lesions as clusters of small round dark grey spots surrounded by circular whitish depigmented zones at the macula. ARPE, also known as Krill's disease, resolves spontaneously over weeks to months.^[1]^ Trial of oral steroids in few cases demonstrated a variable response.^[3]^

**Figure 1:**
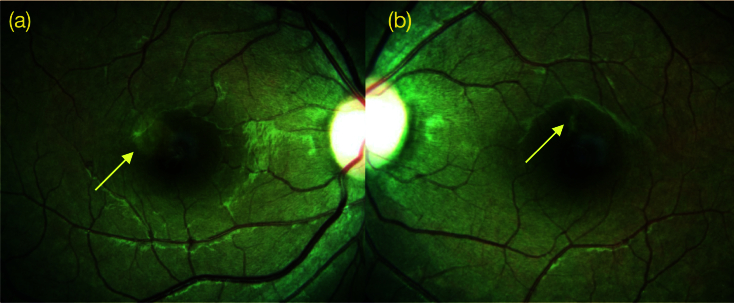
(a) Fundus photograph of right eye showing a cluster of speckled white dots in the parafoveal region. (b) Fundus of the left eye showing a linear whitish lesion superior to the fovea in the parafoveal region.

**Figure 2:**
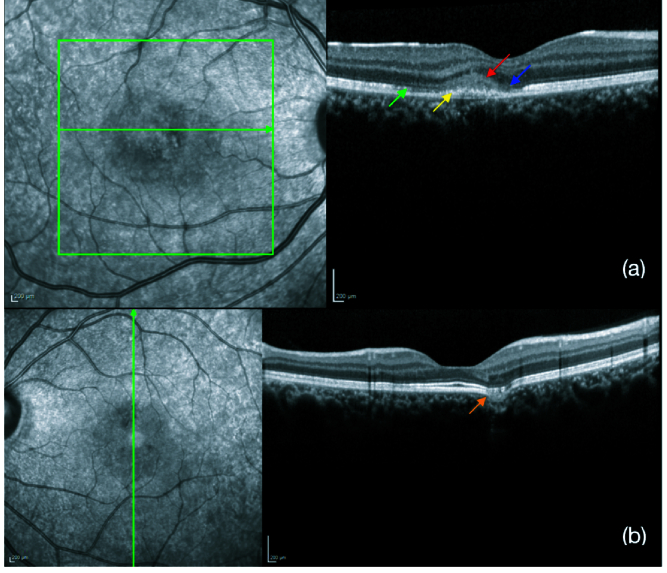
(a) Spectral domain optical coherence tomography (SD-OCT) through the lesion showing abnormal reflectivity of the retinal pigmentary epithelium (RPE) (yellow arrow), disruption of the ellipsoid zone (green arrow) and external limiting membrane (blue arrow), and hyper-reflectivity of the outer nuclear layer (red arrow). (b) SD-OCT through the lesion in left eye showed outer retinal layers conforming to the RPE and excavating into the choroid suggestive of focal choroidal excavation (orange arrow).

**Figure 3:**
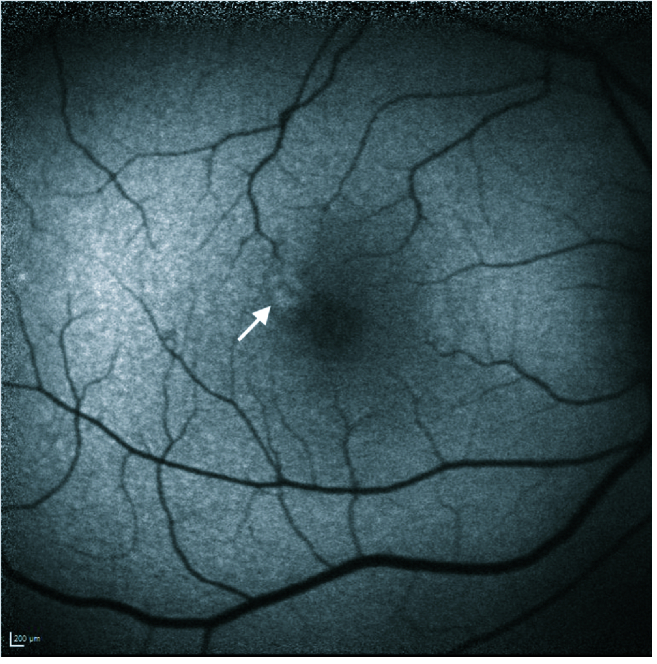
Fundus autofluorescence of the right eye showing increased fluorescence at the area of the lesion suggestive of inflammation of the retinal pigmentary epithelium (white arrow).

**Figure 4:**
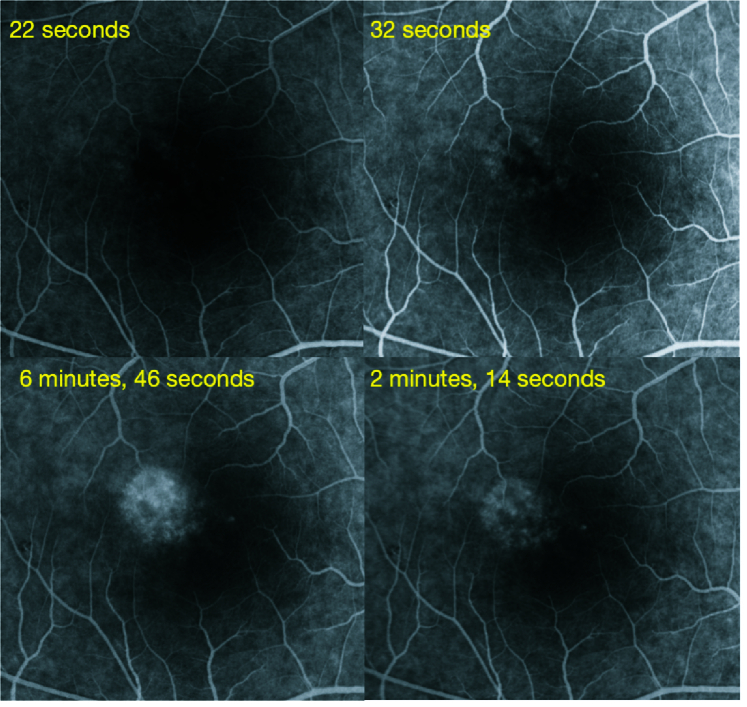
Fundus fluorescein angiogram of the right eye at presentation showing transmitted patchy hyper-fluorescence which increased in later phases without leaking of the dye in the surrounding tissue.

**Figure 5:**
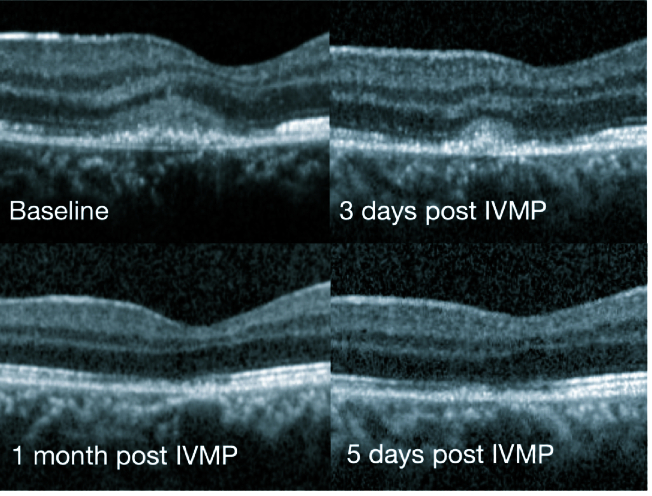
Follow-up spectral domain optical coherence tomography showing resolution of the hyperreflectivity of the outer nuclear layer along with gradual resolution of the external limiting membrane and ellipsoid zone disruptions lesion within five days of Intravenous Methylprednisolone. Minimal disruption of the retinal pigment epithelium is still seen at one month follow-up.

##  CASE REPORT

We report a case of a 22-year-old female who came to us with sudden onset painless blurring of vision in her right eye, with a persistent positive scotoma in the center of the visual field. There was no history of recent fever or any other significant history. Right eye vision was 6/7.5, N8, and left eye was 6/6, N6. Color vision was normal. Anterior segment was normal in both eyes. Intraocular pressure was 14 mmHg in both eyes. Fundus examination in the right eye revealed an area 1/3 disc diameter in size superotemporal to the fovea with retinal pigment epithelial stippling and surrounding pale halo. The left eye showed a linear whitish scar above the fovea [Figure 1]. Spectral domain optical coherence tomography (SD-OCT) through the lesion showed abnormal reflectivity of the retinal pigmentary epithelium (RPE), disruption of the ellipsoid zone, and external limiting membrane and hyper-reflectivity of the outer nuclear layer. Left eye scan through the lesion above fovea revealed an area of focal choroidal excavation [Figure 2]. Fundus autofluorescence of the right eye showed increased fluorescence at the area of the lesion [Figure 3]. Fundus fluorescein angiogram (FFA) of the right eye showed transmitted patchy hyperfluorescence, which increased in later phases without leaking of the dye in the surrounding tissue [Figure 4]. Based on these typical findings, we made a diagnosis of right eye ARPE. We discussed the treatment option with steroids and explained the possibility of spontaneous resolution of the disease over a few months. However, she was keen on rapid resolution of symptoms, and hence we advised Intravenous methylprednisolone (IVMP) 1 gr daily for three days followed by tapering doses of oral steroids. After the first injection, the patient developed gastrointestinal symptoms and discontinued the treatment on her own.

On follow-up, the SD-OCT showed a reduction in the hyperreflectivity and the height of lesion at day 3 and almost complete resolution by day 5 [Figure 5]. The vision had improved to 6/6, N6 and there was a significant subjective reduction in scotoma size. At one month, she was completely asymptomatic and maintained her vision.

##  DISCUSSION 

The diagnosis of ARPE was made based on the characteristic features of acute central scotoma with no sign of any other ocular disease, a solitary, fine stippling in the macular area surrounded by hypopigmented halos, and the presence of a typical hyper-reflective lesion at the outer retina on SD-OCT.^[3]^ Based on the typical FFA findings of ARPE^[2]^ noted in our case and no significant history, we excluded other causes of acute disease, which manifested as stippled pigmented lesions at the macula. These include acute posterior multifocal choroiditis, multiple evanescent white dot syndrome, acute posterior multifocal placoid pigment epitheliopathy, acute macular neuroretinopathy, serpiginous choroiditis, punctate inner choroidopathy, multifocal choroiditis, and laser-induced retinopathy.^[1]^ Although no specific etiology has been found for ARPE, it has been postulated that viral infections and Hepatitis C may be associated.^[5,6]^ ARPE secondary to an intravenous bisphosphonate has also been reported.^[7]^ In our case, we could not find any apparent etiological factor responsible for ARPE.

Previous studies believed that the primary site of inflammation was the retinal pigment epithelium (RPE). Recent studies using spectral-domain optical coherence tomography have suggested that the primary site of inflammation is the interdigitation zone, that is, the contact between photoreceptors and RPE instead of the RPE itself.^[3]^ ARPE being a self-limiting disease, a spontaneous resolution is noted within 6–12 weeks. In a large case series on ARPE, spontaneous recovery was noted in almost 89% of cases within two months.^[1]^

The use of oral steroids has been described in the literature in a series of three patients out of four, however, it did not hasten the recovery.^[3]^ This could have probably been due to a delay in the onset of action of oral steroids. Further, the response to steroids was assessed after a delay of one month. High-dose intravenous steroid treatment is a form of steroid therapy recommended as an initial treatment in optic neuritis and other serious inflammatory ocular diseases.^[8]^ We gave a trial of IVMP in anticipation of a rapid anti-inflammatory response. With a single dose of IVMP, we saw prompt resolution of the hyperreflectivity within five days, associated with a significant symptomatic improvement. Using IVMP as a treatment modality could offer a new way of treating cases of ARPE. Being a rare disease, it may be difficult to study a specific treatment modality in a prospective controlled trial. To the best of our knowledge, this is the first case report highlighting the possible beneficial effect of intravenous steroids in Krill's disease.

##  Financial Support and Sponsorship

None.

##  Conflicts of Interest

None.
